# In silico prediction, characterization and molecular docking studies on Glutathione-S-transferase as a molecular sieve for toxic agrochemicals explored in survey of North Indian farmers

**DOI:** 10.1016/j.heliyon.2021.e07875

**Published:** 2021-08-26

**Authors:** Aggarwal Ritika, Gera Ritika, Jain Nikita, Kaur Bableen, Murali Arunima, Baruah Minakshi, Supriya Anu, Atre Nitin, Khedkar Dinesh

**Affiliations:** a Department of Biosciences and Bioengineering, Indian Institute of Technology Bombay, Powai, Mumbai, Maharashtra, 400076, India; b Department of Biotechnology, Ambala College of Engineering and Applied Research, Devsthali Ambala, Kurukshetra University, Kurukshetra, Haryana, 133101, India; c Department of Chemistry, JAV College, CCS University, Meerut, Uttar Pradesh, 250611, India; d Department of Biotechnology, Jamia Millia Islamia University, Okhla, Delhi, 110025, India; e Department of Biotechnology, St. Thomas College, Ruabandha Bhilai, Hemachand Yadav University, Chattisgarh, 490009, India; f Department of Biotechnology, Gauhati University, Guwahati, Assam, 781030, India; g Department of Chemistry, Central University of Haryana, Jant-Pali, Mahendragarh, Haryana, 123031, India; h Bioinformatics and Data Management, ICMR - National Institute of Virology, Pune, India; i Dept of Botany, Shri Shivaji Science College, Amravati, Sant Gadgebaba Amravati University, Amravati, India

**Keywords:** Glutathione transferase, Bordeaux mixture, Indoxacarb, Agrochemical, Molecular docking

## Abstract

All across the globe, India is considered as an agricultural nation because its agro products drive the economy. An increase in population growth and a hike in food demands lead to the use of hazardous chemicals in farm fields. An in-depth field survey in Northern India was conducted to understand the types of agrochemicals that were used, farmers' knowledge about their safe handling, and their practices on its usage. Ninety-two responders (primarily farmers) from 37 districts of 12 states were interviewed to collect the information. The library containing 58 compounds as toxic spray constituents were developed and further screened *in-silico* for ADMET, drug-likeness, toxicity prediction, and molecular docking against their target actions in the human system. Glutathione S-transferases (GSTs) was selected as target protein showing the best-docked score with Bordeaux, Indoxacarb, Cyphenothrin, Deltamethrin, and Beta-cyfluthrin. The study revealed various adverse effects on human health and advocated provisions of alternative solutions such as using GST as a binding agents to hold the toxic chemicals out of living system and eventually saves valuable lives of the farmers.

## 1 Introduction

In India, a large population depends on agriculture and allied industries, for it is the population's primary source of livelihood. For better growth and production of crops use of agrochemicals seems to be unavoidable. Although many of them are shown to be efficacious on crop production, they are hazardous for the environment and farmers due to occupational exposures (Gupta, 2004). According to government data, more than 500 farmers died due to exposure to toxic agrochemicals in the economic session 2013–2014 and 2017–18 in Maharashtra and Punjab because of the inadequate knowledge about and unavailability of safety measures, posing a high health risk to the farmers (Damalas and Eleftherohorinos, 2011).

The harmful effects of chemicals in use; such as fungicides, insecticides, herbicides; show chronic symptoms/reactions in farmers which sometimes have become lethal. Exposure to such harmful agrochemicals may be through contact with the skin, or ingestion and inhalation. The type of chemical, the duration and route of exposure, and the individual health status determine the possible harmful health outcome (Nicolopoulou-Stamati et al., 2016). The health effects may also vary with the type of cidal chemicals used, for example, organophosphates and carbamates affect the nervous system. Similarly, carbamates can bring about neurotoxicity in the affected subjects while others may irritate the skin or eyes (Sarwar, 2015).

The human body possesses regulatory mechanisms which are self-healing. A healthy body is capable of eliminating the toxic substances generated by its normal functioning and imposed on it by an unnatural lifestyle. Glutathione transferases also referred to as Glutathione S-transferases (GSTs), are ubiquitous and promiscuous enzymes (Markus et al., 2018), shape a collection of multi-gene isoenzymes concerned in the cellular detoxification of xenobiotic and endobiotic compounds (Salinas and Wong, 1999). They were originally termed ligandins because of their ability to bind large molecules, possibly for storage and transport roles (Oakley et al., 1999). Glutathione S transferase (GSTs) is known to be the detoxifying enzyme that catalyzes the glutathione (GSH) conjugation reactions (Fisher, 2001).

But if ingestion of toxic substances overwhelms the detoxification capacity and excretion system then it may be absorbed by the human circulatory system and can undergo various chemical ligand interaction in the body. Thus it can be linked with various deadliest diseases, including cancer, hormone disruption, respiratory diseases like asthma, allergies, and hypersensitivity (Kim et al., 2017).

With a baseline survey in Northern India, the current study collected information from farmers, agriculture shopkeepers, government officials, and others concerning stakeholders' for assessing the impact of agrochemicals on their health. Computational chemistry, particularly virtual screening, ADMET, toxicity prediction, and docking, used to provide valuable insights in finding a hit and lead effective compounds and provides a way to use these binding compounds to further screen and absorb hazardous compounds outside the body itself (Banik et al., 2020). In-silico screening, docking studies, and significant health impacts reveal the urgent need for implementing alternative solutions such as using a trapping agent against the cidal chemicals that is biodegradable with the potential of being reusable and advantageous over the currently used ones.

## 2 Methods

**Figure 1:**
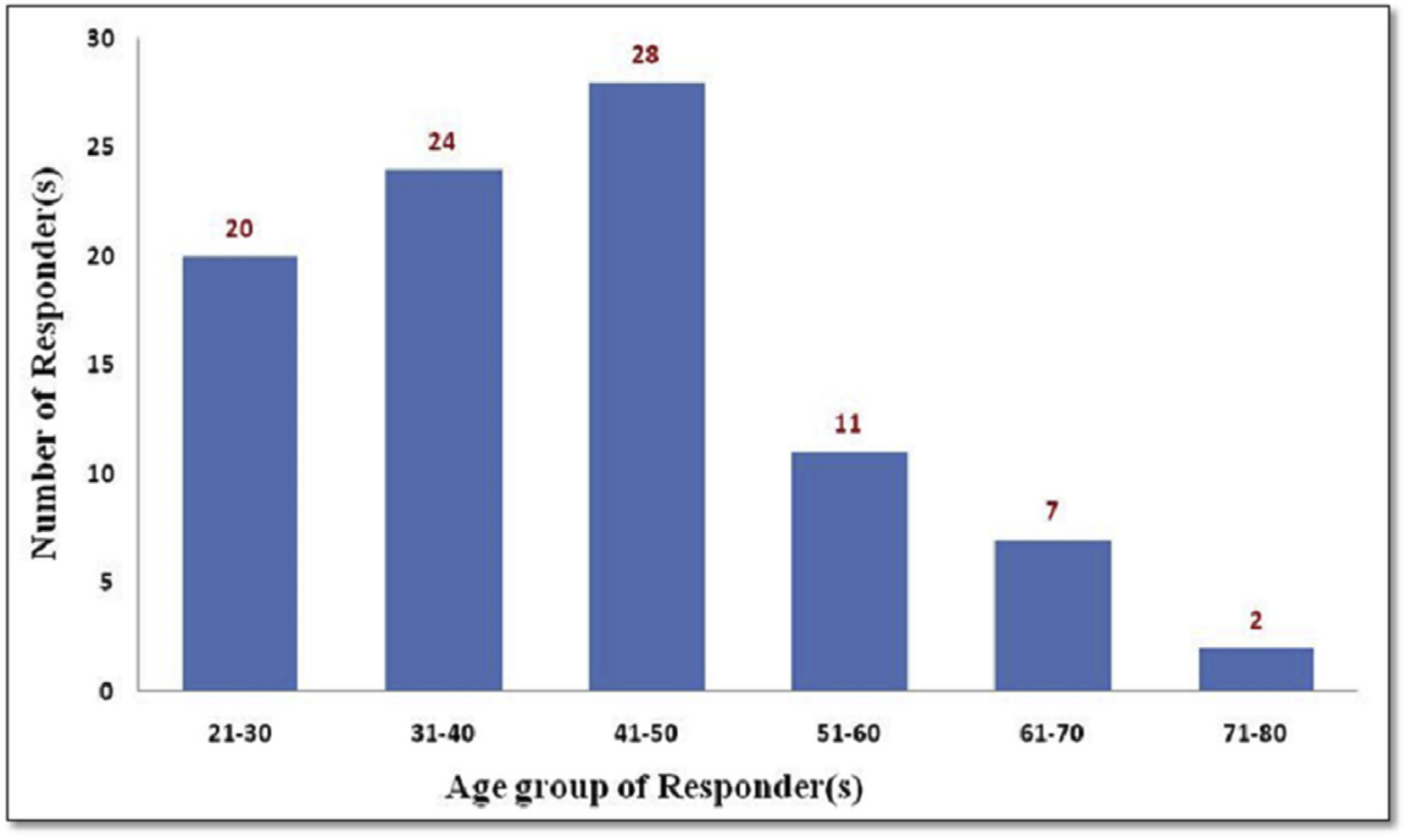
Plan of work to conduct docking studies.

A specific action plan was designed to understand the prevalence of cidal agricultural spray usage, its constituent and impact on farmers’ health (Figure 1). It was executed in Summer Research Training Program – 2020, conducted by the North East Institute of Science and Technology, Jorhat, Assam, India, under an annual program of the Council of Scientific and Industrial Research, a premier R&D organization of India. The current study includes a questionnaire from various responders mainly farmers, statistical as well as chemical analyses of these reported cidal agrochemicals in order to find the most prominent and harmful sprays used in the northern region of India. These chemicals were analyzed for physical, biological and other related properties. Further, these chemicals were docked to find a potential binding target that can be exploited to trap these harmful cidal chemicals outside and hence proposed a way to protect farmers while spraying.

### 2.1 Baseline survey

**Figure 2:**
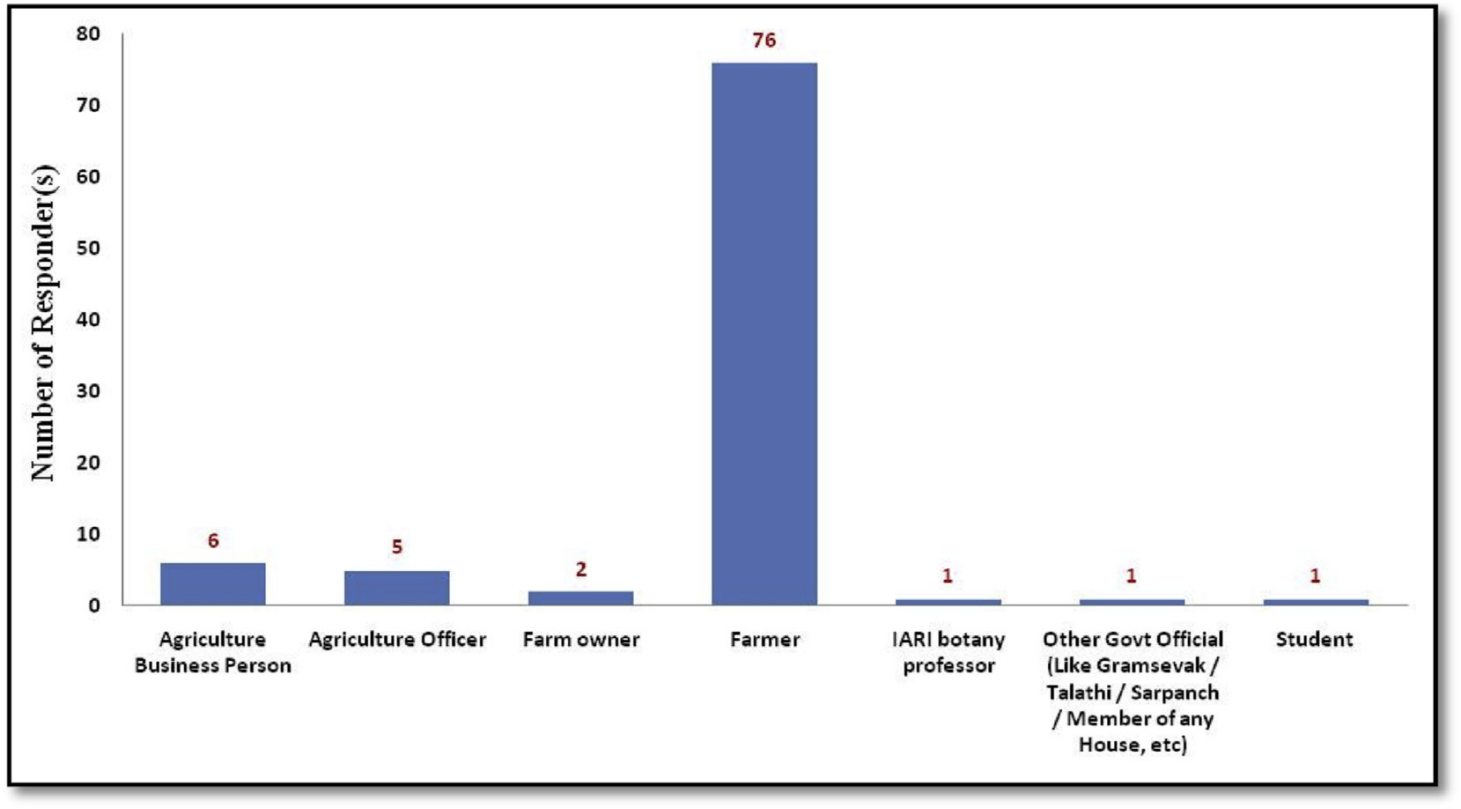
Coverage of the baseline survey northern in India.

**Graph 1:**
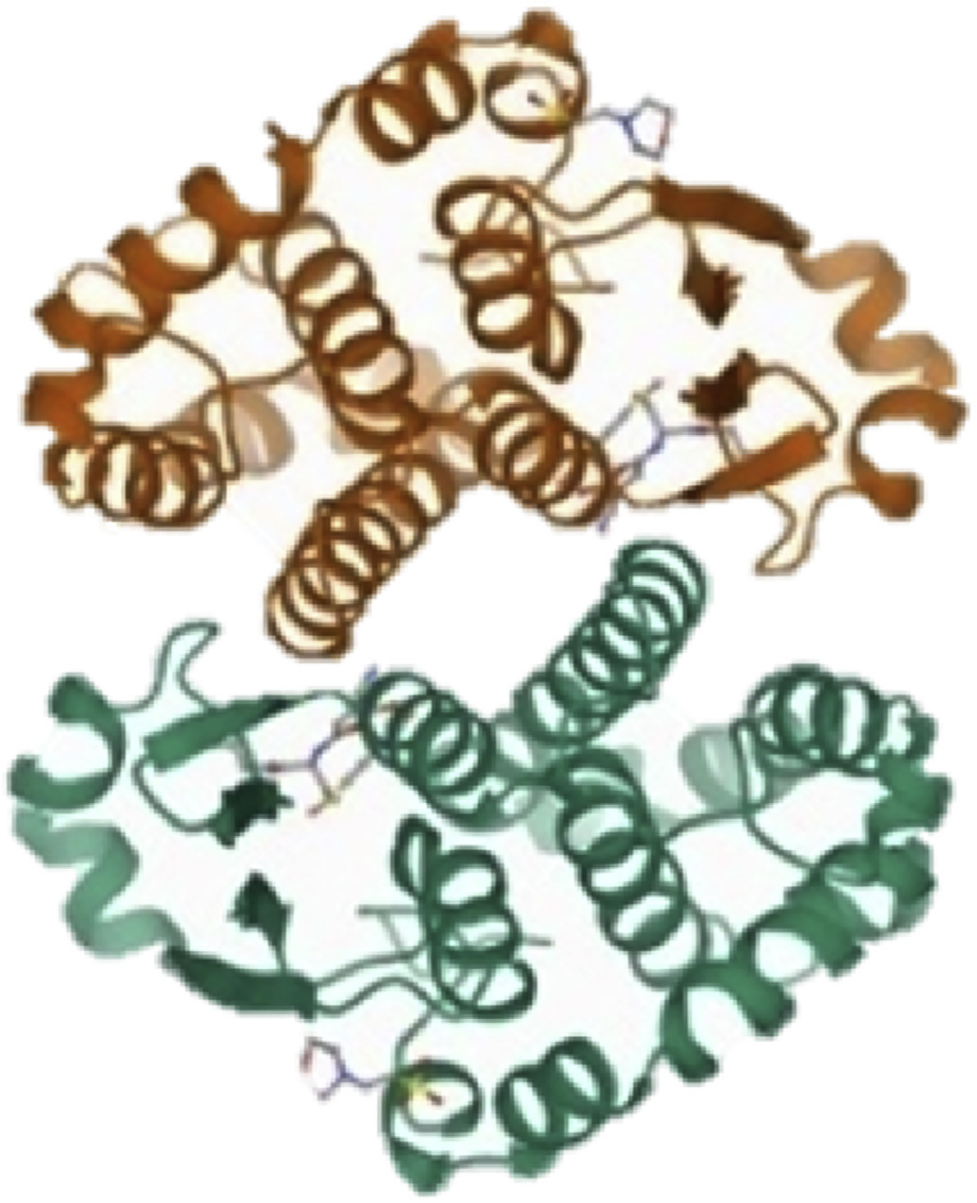
Age group of responders participated in survey.

**Graph 2:**
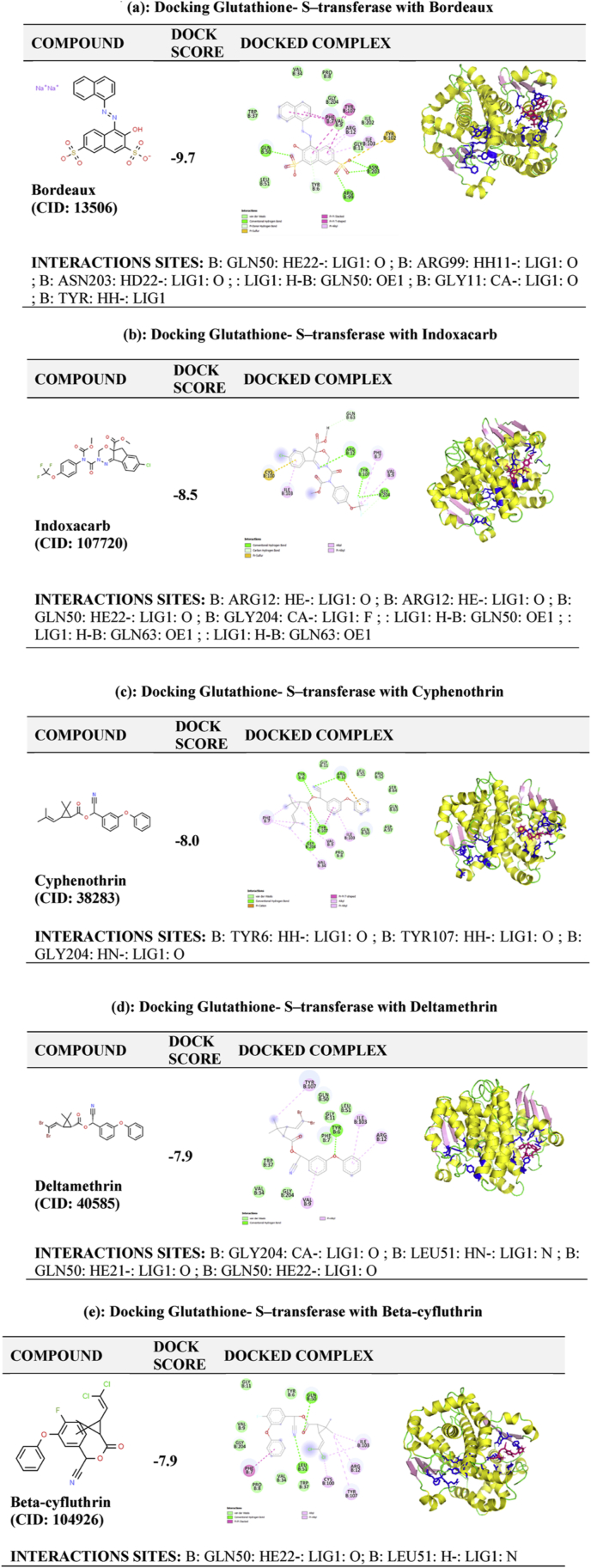
Occupation of the responders participated in survey.

Almost entire Northern India was covered in the baseline survey. The surveyed regions include Assam, Bihar, Chattisgarh, Delhi, Haryana, Himachal Pradesh, Jammu & Kashmir, Jharkhand, Punjab, Uttar Pradesh, West Bengal. During the survey, 37 districts from 12 states were surveyed and the responses of farmers were recorded (Figure 2). Information regarding the places, types of crops, type of chemicals used, pre-and post-health conditions of farmers, area of the agrochemical application, precautions/safety measures taken while spraying, casualties observed, and harmful impact awareness status of the farmer was recorded through the questionnaire. The local farmers, students from agriculture college/university, IARI botany professors, shopkeepers/business personals (agriculture products sellers), agriculture officers, authors/press reporters (agriculture background), contractual workers in fields, farm owners, relatives to farmers, and vegetable suppliers, etc. were interviewed. Responses of 92 respondents from 37 districts of 12 states of India were collected (Graph 2). Most of the respondents interviewed were from the age group of 41–50 years. The ages of the respondents ranged between 21 to 80 (Graph 1). From the baseline survey information, data mining and literature search was performed to discover the most common agrochemicals (toxic spray constituents) used in this region and to be included in the chemical library (Table 1) for further screening.

**Table 1:** The chemical library of the Cidal Spray Constituents.

Sr. No.	Chemical name	PubChem CID	Types	Chemical Safety
1	Acephate	1982	Insecticide	Irritant
2	Acetic acid	176	Herbicides	Flammable, Corrosive
3	Aluminium phosphide	30332	Pesticide	Flammable, Acute toxic, Environmental hazard
4	Atrazine	2256	Herbicides	Irritant, Health hazard, Environmental hazard
5	Benzene hexachloride	727	Insecticide	Irritant, Health hazard, Environmental hazard, Acute toxic
6	Beta-cyfluthrin	104926	Insecticide	Acute toxic, Environmental hazard
7	Bispyribac-sodium	23682789	Herbicides	Irritant, Health hazard, Environmental hazard
8	Bordeaux	13506	Fungicide	Irritant
9	Buprofezin	50367	Insecticide	Health hazard, Environmental hazard
10	Captan	8606	Fungicide	Corrosive, Irritant, Health hazard, Environmental hazard, Acute toxic
11	Carbaryl	6129	Insecticide	Irritant, Health hazard, Environmental hazard
12	Carbendazim	25429	Fungicide	Health hazard, Environmental hazard
13	Carbofuran	2566	Insecticide	Acute toxic, Environmental hazard
14	Carboxin	21307	Fungicide	Irritant, Health hazard, Environmental hazard
15	Cartap hydrochloride	30913	Insecticide	Irritant, Environmental hazard
16	Clodinafop-Propargyl	92431	Herbicides	Irritant, Health hazard, Environmental hazard
17	Cymoxanil	5364079	Fungicide	Irritant, Health hazard, Environmental hazard
18	Cypermethrin	2912	Insecticide	Irritant, Environmental hazard
19	Cyphenothrin	38283	Insecticide	Irritant, Environmental hazard
20	Deltamethrin	40585	Insecticide	Acute toxic, Environmental hazard
21	Dichlorvos	3039	Insecticide, Pesticide	Irritant, Acute toxic, Environmental hazard
22	Dimethoate	3082	Insecticide, Acaricide	Irritant
23	Diuron	3120	Herbicides	Irritant, Health hazard, Environmental hazard
24	Etofenprox	71245	Insecticide	Environmental hazard
25	Fenpropidin	91694	Fungicide	Corrosive, Irritant, Health hazard, Environmental hazard
26	Fipronil	3352	Insecticide	Acute toxic, Health hazard, Environmental hazard
27	Fluchloralin	36392	Herbicides	Environmental hazard
28	Glyphosate	3496	Herbicides	Corrosive, Environmental hazard
29	Hexaconazole	66461	Fungicide	Irritant, Environmental hazard
30	Imidacloprid	86287518	Insecticide	Irritant, Environmental hazard
31	Indoxacarb	107720	Insecticide	Irritant, Health hazard, Environmental hazard, Acute toxic
32	Isoproturon	36679	Herbicides	Health hazard, Environmental hazard
33	Lambda-Cyhalothrin	6440557	Insecticide	Irritant, Environmental hazard, Acute toxic
34	Malathion	4004	Insecticide	Irritant, Environmental hazard
35	Mancozeb	3034368	Fungicide	Irritant, Health hazard, Environmental hazard
36	Metalaxyl	42586	Fungicide	Irritant
37	Methomyl	5353758	Insecticide	Environmental hazard, Acute toxic
38	Metribuzin	30479	Herbicides	Irritant, Environmental hazard
39	Metsulfuron-methyl	52999	Herbicides	Environmental hazard
40	Monocrotophos	5371562	Insecticide	Health hazard, Environmental hazard, Acute toxic
41	Paraquat	15939	Herbicides	Corrosive, Irritant, Health hazard, Environmental hazard, Acute toxic
42	Parathion	991	Insecticide	Health hazard, Environmental hazard, Acute toxic
43	Pendimethalin	38479	Herbicides	Irritant, Environmental hazard
44	Permethrin	40326	Insecticide	Irritant, Environmental hazard
45	Phorate	4790	Insecticide, Acaricide	Environmental hazard, Acute toxic
46	Phosphamidon	3032604	Insecticide, Nematicide	Health hazard, Environmental hazard, Acute toxic
47	Pretilachlor	91644	Herbicides	Irritant, Environmental hazard, Acute toxic
48	Profenofos	38779	Insecticide	Irritant, Environmental hazard
49	Propineb	6100711	Fungicide	Irritant, Health hazard
50	Pyrazosulfuron-ethyl	91750	Herbicides	Irritant
51	Pyriproxyfen	91753	Insecticide	Environmental hazard
52	Quinalphos	26124	Insecticide	Irritant, Environmental hazard, Acute toxic
53	Rotenone	6758	Insecticide	Irritant, Environmental hazard, Acute toxic
54	Tebuconazole	86102	Fungicide	Irritant, Health hazard, Environmental hazard
55	Thiobencarb	34192	Herbicides	Irritant, Environmental hazard
56	Thiram	5455	Fungicide	Irritant, Health hazard, Environmental hazard
57	Triazophos	32184	Insecticide	Irritant, Environmental hazard, Acute toxic
58	Ziram	8722	Fungicide	Corrosive, Irritant, Health hazard, Environmental hazard, Acute toxic

### 2.2 Compound library

The information received through the baseline survey, list of chemicals, products, and their local brand name, was used to prepare a list of chemicals. Data mining, literature surveys were conducted using PubChem Database (Kim et al., 2019) to prepare the final chemical library including information on structural, physical & chemical properties, toxicity, mutagenicity, and related information.

### 2.3 *In-silico* compound screening

Five most potent and widely used chemicals were selected from the compound library. *In-silico* analysis was conducted using ADMET and, DataWarrior tool to have toxicity prediction, and LD50 value (dermal/oral) were documented from PubChem. These studies help in supporting the harmfulness of the substances obtained in the baseline survey. A correlation was made between different chemicals for toxicity and LD50 values. According to the LD50 (oral/dermal) values, these chemicals, categorized in four different classes as per WHO guidelines ("WHO Recommended Classification of Pesticides by Hazard: Guidelines to classification 1990–1991), like -

✓ Class I(a): (LD50 value ≤5 mg/Kg; extremely hazardous)
✓ Class I(b):(LD50 value 5–50 mg/Kg; highly hazardous)
✓ Class II: (LD50 value 50–2000 mg/Kg; moderately hazardous)
✓ Class III: (LD50 value ≥2000 mg/Kg; slightly hazardous)

Drug Likeness Tool (DruLito), an open-source virtual screening tool, was used to calculate each molecule's fast drug-like properties in the compound library. DruLito's calculations are based on the various drug-likeness rules (Table 2A and 2B). Mutagenic, Tumorigenic, Reproductive effective, and Irritant properties were calculated by the DataWarrior tool (Sander et al., 2015)***.***

**Table 2A:** Drug-likeness tool (DruLito) analysis comparative results.

Sr. No.	Filters	Pass	Fail
1	LIPINKSI RULE	44	14
2	GHOSE RULE	42	16
3	CMC RULE	12	46
4	VEBER RULE	50	8
5	MDDR Like RULE	10	48
6	BBB-like RULE	45	13
7	Unweighted QED	42	16
8	Weighted QED	37	21

**Table 2B:** Comparative number of compounds passes the different DruLito filters results.

Sr. No.	Filters pass out of	No. of compounds
1	0 Filters pass out of 8	0
2	1 Filters pass out of 8	1
3	2 Filters pass out of 8	5
4	3 Filters pass out of 8	7
5	4 Filters pass out of 8	11
6	5 Filters pass out of 8	10
7	6 Filters pass out of 8	16
8	7 Filters pass out of 8	8
9	8 Filters pass out of 8	0

### 2.4 Target protein(s) selection and docking studies

**Figure 3:**
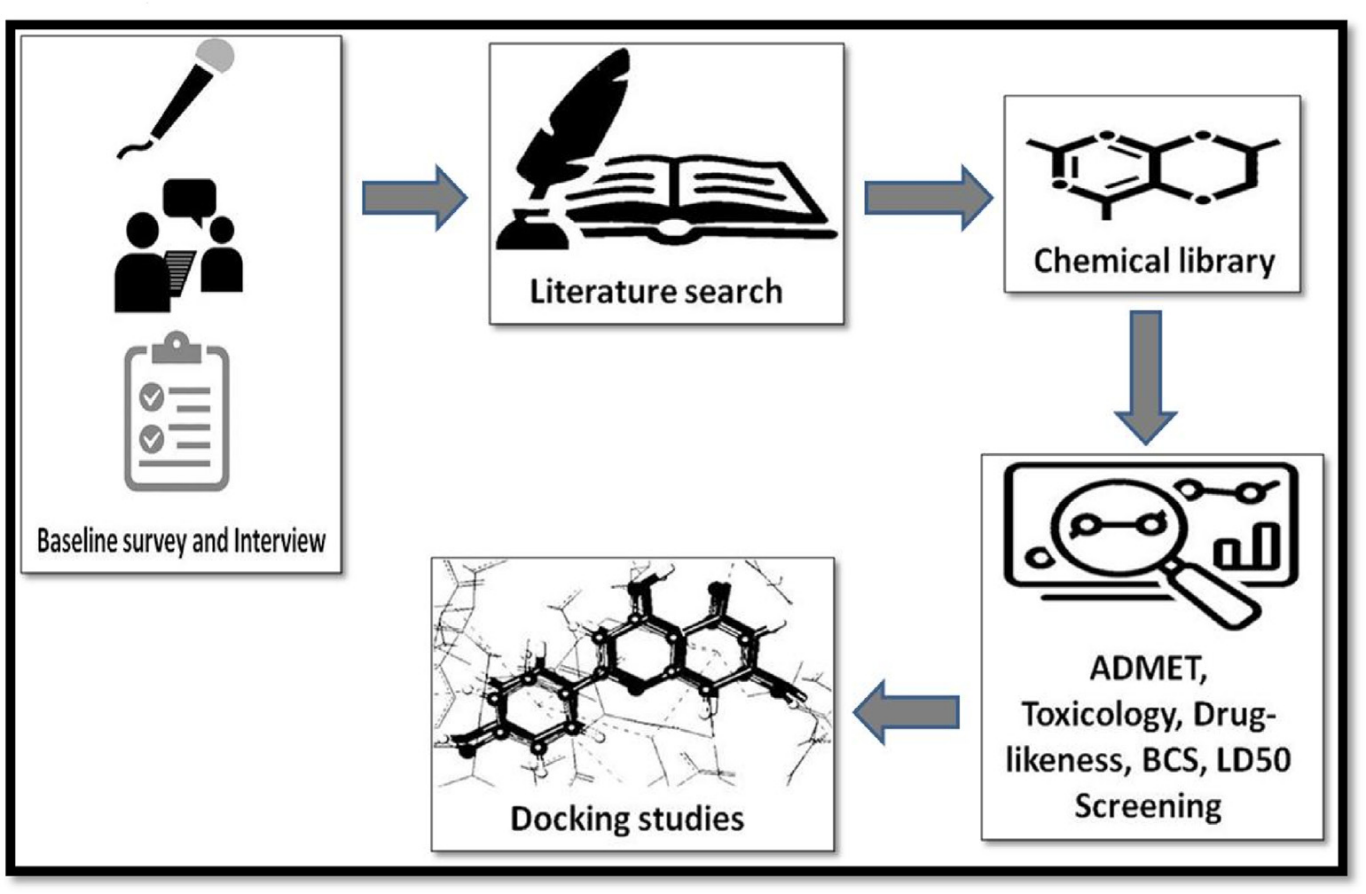
Glutathione s–transferase (PDB ID: 18GS): target protein.

For each compound from the compound library, canonical smiles notation was obtained from the PubChem database (Kim et al., 2019) and was submitted for each compound to predict respective target proteins in the "Swiss Target Prediction" webserver (Gfeller et al., 2014). Based on "known actives (3D/2D)" probability, the relevant target protein was selected and listed for further docking studies. The most common target protein amongst these chemicals was downloaded from RCSB PDB. Glutathione- S–transferase (PDB id: 18GS; Figure 3) selected for further docking studies. Molecular docking studies conducted using Autodock 4.2 (Morris and Lim-Wilby, 2008). Based on their functions, the most common protein and docking diagram were obtained using PyMOL Molecular Graphics System, Version 1.2r3pre, Schrödinger, LLC. The best five docked results were interpreted.

**Figure 4:**
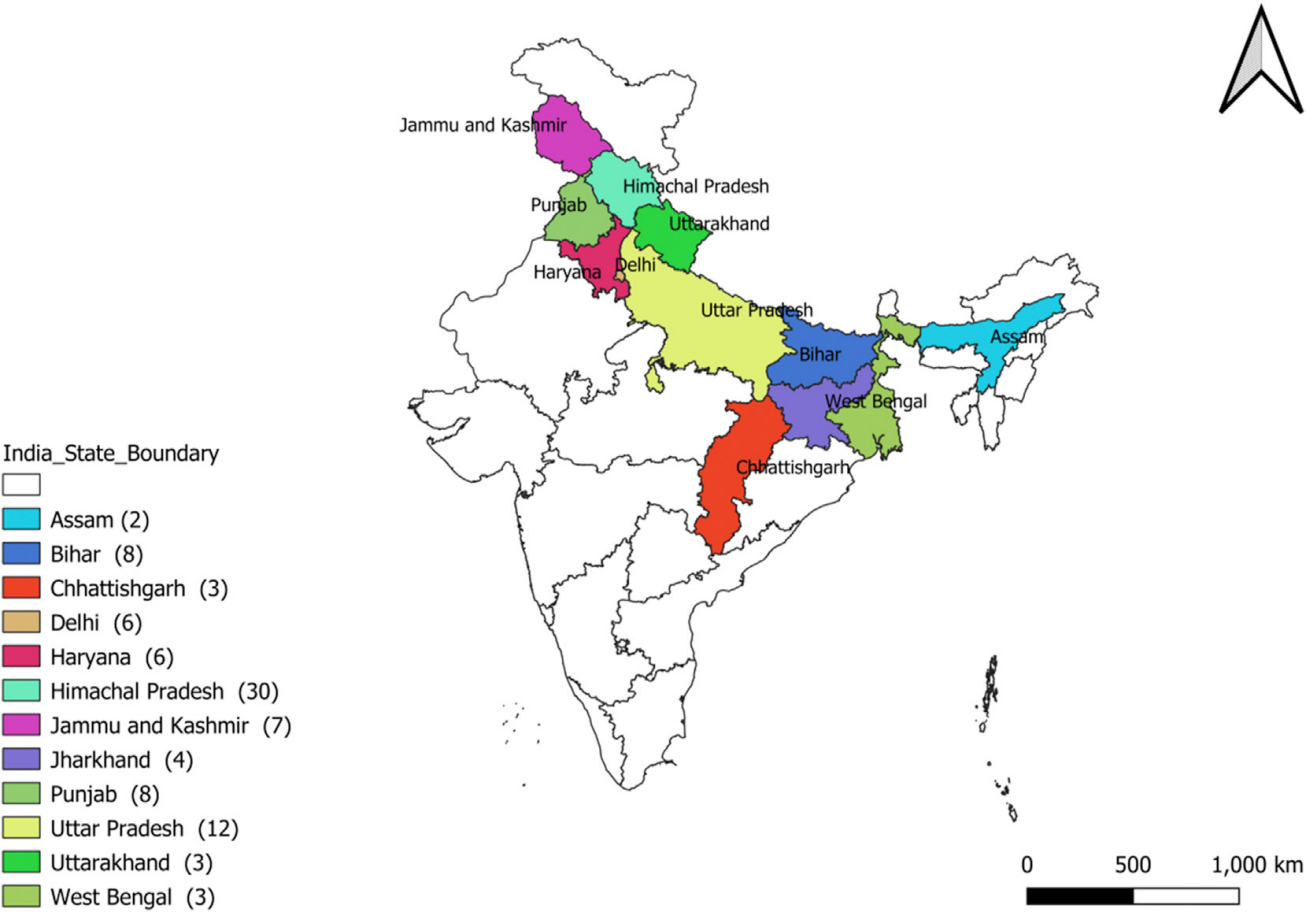
(a, b, c, d, e) Docking of the five selected chemicals with Glutathione – S - transferase. The images on the left side are the docking results obtained from PyMOL. The images on the right side are the 2D view of the interactions between the ligand and binding site of the protein.

The present work is conducted in silico, hence ethical issues were not involved. The baseline survey was initiated under the Summer Research Training Programme under NEIST, Jorhat, Assam, India and the relevant report is submitted (see Figure 4).

## 3 Results

### 3.1 Baseline survey

The statical interpretation of data revealed that most of the respondents interviewed are in 41–50 years age group. The ages of the respondents were ranging between 21 to 80 (Graph 1). In the respondents, primarily farmers were interviewed, i.e.,76 (82.60%), followed by agriculture business persons, agriculture officer, farm owners, IARI Botany Professor, Government officials (Like Gram-Sevak/Talathi/Sarpanch/etc), and student of agriculture (Graph 2).

In the area under study, maximum respondents cultivate Rice crop (26) followed by Wheat (22), Maize (15), Cotton (14), Potato (13), Sugarcane (12), Mustard (11), Vegetables (9), Sorghum (8), Tomato (8), etc. Types of agrochemical sprays used in the field reported by responders were Insecticide (41), Fungicide (26), Herbicide (17), Pesticide (14), Fertilizer (5), Bio-fertilizer (5), weedicide (2), and rodenticide (1).

The questionnaire focusing on the users' safety majors revealed 53.57% of respondents covered their face with cloth/mask, eyes with goggles, and gloves during spray. They also used foot cover/shoes, full sleeve shirt, or any protector. 30.91% of respondents claimed that they did not take any safety precautions. 10.71% of the respondents reported that they avoid direct contact during spray, also avoid eating or drinking during the period of spraying, and leaving the field as soon as possible after spray; The 4.76% of the respondents stated that they do not know about any safety measure for any sprays they used in their farms.

A piece of information seeking the health issues aroused in the agrochemical users exposed that most respondents faced skin problems, including rashes, itching, and dermatitis, followed by eye irritation, and respiratory illnesses including breathlessness, coughing or sneezing. Some of them experienced liver or kidney issues. Headache, nausea, vomiting and muscle cramps, the declining fertility rate in men, loss of appetite, prostate cancer, hair loss, ulcers were also severe ailments in the agrochemical users. Few incidences of death were also reported.

The survey responses to the query "harmful impact awareness status of the farmer regarding spray" revealed that 38.46% were aware; 40.65% accepted that they had incomplete knowledge, and 19.79% were reported to be unaware of the harmful impact.

Total 58 chemicals, categorized into types based on their usage as - acaricide (2), fungicide (13), herbicides (15), insecticide (29), nematicide (1), and pesticide (2). These compounds are already known for chemical safety as of acute toxic (22), corrosive (6), environmental hazard (52), flammable (3), health hazard (24), and irritant (39), as shown in Table 1.

### 3.2 In-silico compound screening

All the compounds from the library were screened for structural, functional, physical, and toxicological parameters. LD50 (oral/dermal) values reported for each compound from PubChem (Kim et al., 2016).

Drug Likeness Tool (DruLito) studies indicated that out of 8 different drug-likeness rules/filters, 16 compounds passed in 6 filters, 11 compounds passed in 4 filters, 10 compounds passed in 5 filters, 9 compounds passed in the highest 7 filters.

The distribution of 58 compounds for mutagenic nature was 31 – high, 4 – low, and 23 – non-mutagenic. Tumorigenic character evaluation revealed that 27– high, 2– low, and 29 were non-tumorigenic. Reproductive effectiveness showed 39– high, 3– low, and 16– non-reproductive effective. For irritant properties, 26– high, 5– low, and 27 were non-irritant. Remarkably, 18 out of 58 (31.03%) were highly mutagenic, tumorigenic, reproductive effective, and irritant in their effects; eventually, they are most dangerous to human and environmental health. At the same time, 11 out of 58 (18.96%) were none for all. However, 29 out of 58 (50%) show at least one or more as high (either mutagenic/tumorigenic/reproductive effective/irritant).

### 3.3 Target protein(s) selection and docking studies

The best five docked complexes were selected based on docking score or the binding capacities. Docking analysis showed that the compound bind with the interacting sites (LIG1, GLN50, ARG99, ASN203, ARG12, GLY204, OE1, TYR6, LEU51) of target protein GSTs (Fereidoonnezhad et al., 2018).

Interaction strength of Hydrogen bond was highest, followed by Vander Waals interaction, pi-pi bond, alkyl, and n-alkyl interactions. (Table 3 Ligands with a greater number of H bond interaction with GST are trapped more easily within the protein, but aromatic interactions at the ligand-protein surface allow the ligand to more strongly bound to the protein and these aromatic interactions were the pi-pi stacking. T-shaped interactions were also found between chemical and protein, which plays a vital role in biological recognition and the organization of biomolecular structures. These interactions have been recognized as one of the key constituents of Ligand-protein interface even though they are found to be weaker than the hydrogen bonding present in ligand-protein interfaces (Brylinski, 2018). These pi-pi stacking interactions form between the aromatic ring-like benzene dimer and the aromatic amino acids (Phenylalanine, Tyrosine, Histidine, and Tryptophan). Among the five selected chemicals, only three chemicals showed the pi-pi interaction, which accounts for their high binding capacity, while the other two had strong hydrogen bonding and van der Waal interactions. Bordeaux showed maximum pi-pi interaction (stacking and T-shaped) between the benzene dimer and aromatic amino acid, Phenylalanine, and tyrosine.

**Table 3:** Docking parameters of the docked complexes obtained from the docking of Glutathione-S-transferase with the selected chemicals along with the number of different types of binding interactions.

Docked complex	Best docking score	No. of conventional H-bond	Van der waals int	Pi-stacked int	Pi-alkyl int	Other attractive interactions∗
Bordeaux	-9.7	4	8	2	2	1
Indoxacarb	-8.5	4	0	0	1	3
Cyphenothrin	-8	4	8	0	2	2
Deltamethrin	-7.9	1	7	0	4	0
Beta-Cyfluthrin	-7.9	2	7	1	2	2

∗Alkyl, pi-sulphur, pi-carbon interactions.

Stronger binding to GST to chemicals (Bocedi et al., 2019) may increase GST levels in the blood, implying a more toxic effect of the chemicals. More in-vitro studies are required to confirm these toxic effects.

Order of binding capacity/docking score among the rest of the chemicals can be seen here i.e. Deltamethrin (-7.9) > Fipronil (-7.6) > Lambda-Cyhalothrin (-7.5) > Metsulfuron-methyl (-7.5)> Permethrin (-7.3) > Fenpropidin (-7.2) > Pyriproxyfen (-7.1) > Buprofezin (-7.0) > Rotenone (-6.9) > Carbaryl (-6.8) > Etofenprox (-6.8) > Carboxin (-6.7) > Pyrazosulfuron-ethyl (-6.6) > Tebuconazole (-6.6) > Imidacloprid (-6.5) > Isoproturon (-6.5) > Carbendazim (-6.4) > Triazophos (-6.4) > Fluchloralin (-6.3) > Pendimethalin (-6.3) > Hexaconazole (-6.2) > Quinalphos (-6.0) > Carbofuran (-5.9) > Clodinafop-Propargyl (-5.9) > Captan (-5.8) > Diuron (-5.8) > Metalaxyl (-5.8) > Metribuzin (-5.8) > Thiobencarb (-5.8) > Paraquat (-5.7) > Profenofos (-5.7) > Parathion (-5.5) > Pretilachlor (-5.5) > Atrazine (-5.4) > Phosphamidon (-5.2) > Cymoxanil (-5.0) > Glyphosate (-4.8) > Malathion (-4.7) > Monocrotophos (-4.6) > Methomyl (-4.5) > Benzene hexachloride (-4.4) > Dichlorvos (-4.3) > Acephate (-4.0) > Dimethoate (-3.9) > Mancozeb (-3.9) > Phorate (-3.9) > Thiram (-3.5) > Propineb (-3.4) > Acetic acid (-3.1) > Ziram (-2.6) and three were not docked to target protein i.e. Aluminium phosphide, Bispyribac-sodium, and Cartap hydrochloride.

## 4 Discussion

Across the world, the use of different kinds of synthetic pesticides for crop protection and reduction of crop damages due to pests, insects, diseases, and weeds is alarming concern about the ill-effects of these agrochemicals on human health (Chand and Birthal, 1997). However, many pesticides have been associated with human health and environmental issues, and they are being used to cope with the increased food demands. More commonly, farmers, their families, and co-workers are at a higher risk of being affected (Sapbamrer and Nata, 2014). The toxicity of chemicals depends on the toxicant's nature, exposure routes (oral, dermal, and inhalation), dose, and organism. Consistent and constitutive exposure to sub-lethal quantities of pesticides for extended periods causes intense chronic infections in humans (Asghar et al., 2016). Recently several studies establish a link between pesticide exposure and the incidences of human chronic diseases like cancer (Xu et al., 2010). Many studies confirm pesticide residues in food commodities, groundwater, ingesting water, bottled water, and many others (Tyagi et al., 2015). Parallel to these published reports, undertaken survey also evident many subjects suffering from these reported diseases.

The chemicals that are screened during the survey are hazardous and based on their LD50 values, and they are classified into four different categories. Among the five selected chemicals based on their docking score, Beta-cyfluthrin, and Cyphenothrin are moderately hazardous, while Deltamethrin is highly hazardous. Deltamethrin found to be lethal when breathed in. Individuals who have ingested a large amount of Deltamethrin experienced nausea, vomiting, abdominal pain, and dizziness. Among the selected five chemicals, Beta-cyfluthrin, Deltamethrin, and Cyphenothrin are pyrethroid, and indoxacarb is Oxadiazine pesticide. Other studies reported the reproductive effect of pyrethroid and other pesticides also (Abbassy et al., 2014).

As these chemicals are highly toxic when inhaled, it is necessary to prevent these chemicals from entering an individual's body. For that purpose, an attempt was made to determine which proteins can bind such chemicals outside the body. GSTs were used as a target protein in the current study to identify its prominent binding sites with 58 chemicals documented and *in-silico* screened. GSTs are multi-functional detoxification iso-enzymes and have a crucial role in cellular signalling (Özaslan et al., 2018). Among the five chemicals, three were binding more strongly to the target protein and shows aromatic interaction plays an essential role in the ligand-protein interface. Hydrogen bonding between the ligand and target protein increases the binding strength and increases the chances of being trapped.

Insecticides can function by impeding acetylcholinesterase, blocking different voltage-gated ion channels, hampering different metabolic pathways, and targeting essential proteins involved in respiration. Herbicides can affect aromatic amino acid biosynthesis and carotenoid formations (Casida and Durkin, 2017). Dithiocarbamate is seen to be potent inhibitors of other development-related cell signalling pathways (Wei et al., 2021).

Several published reviews are inconsonance like - there were indications of adverse effects in users of Bordeaux mixture that were exacerbated by smoking, and it is harmful if inhaled (Arena et al., 2018). Methemoglobinemia occurred to a human patient following Indoxacarb's ingestion and an oxadiazine pesticide used to control cotton bollworm, budworm in cotton, and soybeans (Prasanna et al., 2008). Several studies documented Cyphenothrin, Fenpropathrin as causal elements of both the T and CS syndromes (Soderlund et al., 2002). The primary symptoms of intoxication with Cyphenothrin (WHO Acute Hazard classification: Class II, moderately hazardous) and other synthetic pyrethroids affect mainly the nervous and muscular systems. The most frequent symptoms are Ataxia, Hyperreactivity, Tremor, Paresthesia, Exhaustion, and Hypersalivation (Junquera, 2017). A case of a 32-year-old woman admitted to the emergency department (ED) with irritability, muscle cramps, discomfort, and sensation of burning, loss of sensation in her feet and arms, and dyspnea due to deltamethrin ingestion (Gunay et al., 2010). The study also reveals that higher doses of deltamethrin ingestion may cause severe symptoms. Few studies show cyfluthrin and beta-cyfluthrin are moderate anti-androgenic chemicals (Zhang et al., 2008).

Pyriproxyfen toxicity is known to decrease fertility in women (Plumb, 2015). Hexaconazole showed slight to moderate acute oral toxicity in rats and mice (Worthing and Hance, 1991). Fipronil is acutely toxic in humans, as it causes DNA damage and is also known to cause neuroblastoma (Vidau et al., 2011). Dichlorvos exerts its toxic effect by irreversibly inhibiting neural acetylcholinesterase. The inhibition provokes the accumulation of acetylcholine in synapses with disruption of nerve function. It also damages the liver, interferes with fatty acid metabolism, and disturbs the antioxidant defence system in rats (Jin et al., 2015). Profenofos can cause cholinesterase inhibition in humans; that is, it can stimulate the nervous system causing nausea, dizziness, confusion, & at very high exposures (e.g., accidents or major spills), respiratory paralysis & death (El-Sebae et al., 1988). Toxicity ranges from mild skin rashes, eye irritation, vomiting, diarrhoea to severe carcinogenic effects. It causes mutation damaging DNA (Lisi et al., 1987).

GST enzymes protect against oxidative stress. Compounds like ROS (reactive oxidant species) and OS (oxidant species) can cause DNA, protein, and lipid damage with the onset of chronic and non-communicable diseases (Wang et al., 2013). Due to exposure to these pesticides, erythrocyte GST in the blood is occupied with trapping these chemicals, making it unavailable for ROS and OS. OS plays a decisive role in reducing cognitive function and the ageing process (Mariani et al., 2005). e-GST has been used as a biological marker for industrial toxins released from chemical industries. Research also showed that important industrial chemicals such as propylene oxide and ethylene dichloride inhibited GST from erythrocytes in situ and purified GST (Gouda et al., 2016). This suggests that chemical exposure results in the reduced capability of e-GST to detoxify xenobiotics, making the body incapable of dealing with metabolic stress. When exposure to 1,3- butadiene (oxidizing compound) was studied (Primavera et al., 2008), it was found that e-GST was impaired in the workers of industrial areas, which suggests that the Gst activity levels and the glutathionylated haemoglobin (Srivastava, 1981) levels can be recommended as promising biomarkers. Earlier studies done have established that Glutathione S transferase (GST) is a potential electrochemical transducer to be used as substrate in Biosensors made for pesticide detection due to its strong interactions with pesticides (bendiocarb, DDT, and parathion) (Shahbaaz et al., 2018).

## 5 Conclusion

Docking is a valuable technique to reveal counter actions between the chemicals. The actions of agrochemicals threatening and costing the thousands of lives of food producers around the world. Such toxic agrochemicals devastating metabolic processes in the human system may be arrested before their entry. Docking studies were considered a vital tool and arrived at one biomolecule GST with an affinity towards many toxic agrochemicals. An exhaustive survey in the Northern part of India including, farmers, officers and other involved people in the usage of agrochemicals, revealed many facts and upraised an urgency of tool to trap such killer chemicals out of the body. The survey has also evolved with numerous health disorders due to prolonged contact with the agrochemicals and ignorance of farmers regarding the safety measures.

The docking studies discovered the biomolecule Glutathione S – Transferase as a potential candidate against the maximum chemicals as blocker/inhibitor/chemical screen/adsorbent/absorbent. The significant outcome of the present research is the establishment of the platform to devise any tool to arrest toxic agrochemicals. prior to the direct encounter with the human biological system.

## Declarations

### Author contribution statement

Ritika Aggarwal, Ritika Gera, Bableen Kaur & Nitin Atre: Performed the experiments; Analyzed and interpreted the data; Wrote the paper.

Nikita Jain, Arunima Murali, Minakshi Baruah & Anu Supriya: Performed the experiments; Analyzed and interpreted the data.

Dinesh Khedkar: Conceived and designed the experiments; Wrote the paper.

### Funding statement

This research did not receive any specific grant from funding agencies in the public, commercial, or not-for-profit sectors.

### Data availability statement

The authors are unable or have chosen not to specify which data has been used.

### Declaration of interests statement

The authors declare no conflict of interest.

### Additional information

No additional information is available for this paper.
